# Higher pelvic incidence values are a risk factor for trans-iliac trans-sacral screw malposition in sacroiliac complex fracture treatment

**DOI:** 10.1186/s10195-023-00728-0

**Published:** 2023-09-21

**Authors:** An-Jhih Luo, Angela Wang, Chih-Yang Lai, Yi-Hsun Yu, Yung-Heng Hsu, Ying-Chao Chou, I.-Jung Chen

**Affiliations:** 1 Division of Orthopedic Traumatology, Department of Orthopedic Surgery, Chang Gung Memorial Hospital, No. 5, Fu-Hsing Street, Kweishan 333, Taoyuan, Taiwan; 2 Bone and Joint Research Center, Chang Gung Memorial Hospital, No. 5, Fu-Hsing Street, Kweishan 333, Taoyuan, Taiwan; 3 Department of Orthopedic Surgery, Taipei Veterans General Hospital, No. 201, Sec. 2, Shipai Rd., Beitou District, Taipei City, 11217 Taiwan ROC

**Keywords:** Pelvic fracture, Pelvic incidence, Iliosacral screw, Trans-iliac trans-sacral screw

## Abstract

**Background:**

Percutaneous iliosacral (IS) screw fixation and trans-iliac trans-sacral (TITS) screw fixation are clinically effective treatments of posterior pelvic sacroiliac fractures. In order to accurately assess the sacrum position relative to the pelvis, pelvic incidence (PI) is a commonly utilized radiographic parameter in sagittal view. This study aimed to investigate and compare the surgical outcomes and radiographic parameters of IS or TITS screw fixations for the treatment of posterior sacroiliac complex fractures with different PI values.

**Materials and methods:**

The data on patients with posterior pelvic sacroiliac fractures who underwent percutaneous IS or TITS screw fixations, or both, at a single level I trauma center between January 2017 and June 2020 were reviewed. We documented the patient characteristics and fracture types, reviewed surgical records, and measured the radiographic parameters via plain films and multi-planar computed tomography (mpCT) images. Radiographic variations in PI, sacral slope, pelvic tilt, sacral dysmorphism, pelvic ring reduction quality, screw deviation angles, screw malposition grading, and iatrogenic complications were documented and analyzed.

**Results:**

A total of 85 patients were included, and 65 IS and 70 TITS screws were accounted for. Patients were divided into two groups according to screw fixation method and further divided into four sub-groups based on baseline PI values. The PI cutoff values were 49.85° and 48.05° in the IS and TITS screw groups, respectively, according to receiver operating characteristic analysis and Youden's J statistic. Smaller PI values were significantly correlated with sacral dysmorphism (*p* = 0.027 and 0.003 in the IS and TITS screw groups, respectively). Patients with larger PI values were at a significantly increased risk of screw malposition in the TITS screw group (*p* = 0.049), with no association in the IS screw group. Logistic regression confirmed that a larger PI value was a significant risk factor for screw malposition in the TITS screw group (*p* = 0.010). The post-operative outcomes improved from poor/fair (at 6 months) to good/average (at 12 months) based on the Postel Merle d'Aubigné and Majeed scores, with no significant differences between subgroups.

**Conclusions:**

Both percutaneous IS and TITS screw fixations are safe and effective treatments for posterior pelvic sacroiliac fractures. Due to the higher risk of screw malposition in patients with larger PI values, it is crucial to identify potential patients at risk when performing TITS screw fixation surgery.

***Level of evidence*:**

Level III.

## Introduction

Pelvic fractures constitute about 3–8% of all skeletal injuries and are often caused by high-energy trauma, such as car collisions, falls from height, or crush injuries [1–3]. The posterior sacroiliac complex makes a highly valuable contribution to pelvic stability. As a weight-bearing structure, the vertebral column weight is transmitted laterally through the pelvic girdle and hip joints, and these posterior structures are responsible for approximately 70% of the stability of the pelvic ring, whereas the contribution of anterior structures is smaller [4, 5].

Percutaneous reduction and fixation with iliosacral (IS) screws was first introduced by Vidal et al. in 1973, followed by the trans-iliac trans-sacral (TITS) screw technique of Gardner and Routt in 2011 [6, 7]. Both techniques are minimally invasive and effective procedures to treat stable and unstable posterior pelvic sacroiliac fractures. Furthermore, these methods can be implemented as part of damage control surgery to ensure pelvic stability in an emergency setting or as a timely definitive internal fixation, allowing concomitant surgical procedures to be performed. Considering their many advantages, including excellent biomechanical stability, the achievement of anatomic or near-anatomic pelvic reduction, minimal blood loss, and low infection rate, they have become widely accepted methods [8, 9].

Anatomically, the position of the sacrum with respect to the pelvis varies with the individual, and so does the shape and surface area of the sacroiliac joint. Pelvic incidence (PI), first introduced by Legaye et al. [10], is an important radiographic parameter in spine surgery. Representing the main axis of the sagittal balance of the spine, it is defined as the angle between the line perpendicular to the sacral plate at its midpoint and the line connecting the same point to the center of the bicoxofemoral axis. The PI is constant and unique to each individual, but shows great variability, ranging from 33° to 85° in the normal population [11].

The authors anecdotally experienced difficulties with the insertion of IS and TITS screws in patients with large PI values and recognized that the relative positions of the sacrum and pelvis might have an influence on screw insertion. Therefore, this study aimed to investigate and compare the surgical outcomes and radiographic parameters of IS or TITS screw fixations for the treatment of posterior sacroiliac complex fractures with different PI values. To the best of our knowledge, no previous studies have explored the clinical significance of PI values in the treatment of posterior pelvic sacroiliac fractures with IS and TITS screws.

## Materials and methods

### Patient recruitment

We performed a single-center, retrospective cohort study at a level I trauma center with the approval of the relevant institutional review board. A retrospective chart review was conducted and documented by consecutively reviewing the data of patients with pelvic sacroiliac complex fractures. The inclusion criteria of the study were: (1) patients with pelvic sacroiliac complex fractures with or without an anterior pelvic ring fracture or acetabular fracture; (2) patients who underwent percutaneous IS or TITS screw fixation or both; (3) patients who completed all the comprehensive pre- and post-operative imaging, including radiographs of the anterior–posterior (AP) view and inlet and outlet projections as well as multi-planar computed tomography (mpCT) of the pelvis; and (4) patients who had completed at least 1 year of post-operative follow-up. Patients were excluded from the study if (1) the pelvic sacroiliac complex fracture was associated with pathological conditions such as malignancy; (2) patients lacked the required imaging data; and (3) patients were under 16 years of age. A total of 85 patients were collected and included in this study between January 2017 and June 2020. The data encompassed comprehensive records of each patient’s demographic characteristics, comorbidities, pre- and post-operative radiographic parameters, as well as complications.

### Assessment of images and associated parameters

Plain films of AP, inlet, and outlet projections and mpCT of the pelvis were acquired for preoperative evaluation at the emergency department or after admission. Image assessment and radiographic parameter measurement were conducted via the Picture Archiving and Communication System (Centricity Enterprise Web V3.0; GE Healthcare, Chicago, USA). Radiographic evaluation was performed using the established method in an unbiased fashion by two independent certified surgeons of our orthopedic department who did not participate in the surgeries.

Pelvic sacroiliac complex fractures were further classified using the Arbeitsgemeinschaft für Osteosynthesefragen/Orthopaedic Trauma Association (AO/OTA) classification, Tiles classification, and Young-Burgess classification [12, 13]. Sagittal parameters such as PI, sacral slope, and pelvic tilt were measured using mpCT scanograms based on the method described by Legaye et al. [10]. Furthermore, sacral dysmorphism was defined and counted according to the works of Routt, Carlson, and Kaiser et al. [14–16]. As part of our postoperative protocol, comprehensive assessment of pelvis series, including plain films of the AP, inlet, outlet, and mpCT, were obtained for evaluation. To determine the postoperative reduction quality of the pelvis, the Matta/Tornetta and Lefaivre criteria were applied to evaluate the restoration of the vertical, rotational, and symmetrical anatomy of the pelvic ring with a rating system of excellent, good, fair, and poor results [17–19].

To access and quantify the extent of screw malposition, a grading system proposed by Smith et al. [20] was used to evaluate postoperative mpCTs; in this grading system, grade 0 corresponded to no perforation, grade 1 to a perforation of less than 2 mm, grade 2 to a perforation of between 2 and 4 mm, and grade 3 to a perforation of more than 4 mm. The correlation between the IS and TISS screws and the sacroiliac joint was further analyzed. The angular deviations from ideal orientation (ADIO) of all IS and TITS screws in the axial and coronal planes of mpCT were documented and calculated using the equation from the study by Chen et al. [21]

### Surgical procedure

Surgical planning was conducted with a comprehensive review of the preoperative plain films and mpCT images. Based on the preoperative planning, the surgical position was decided upon based on the fracture pattern, surgical approach, and concomitant injury. The supine position was used if patient had an anterior pelvic ring fracture or the presence of a concurrent intra-abdominal injury. The prone position was chosen in cases with severe spinopelvic dissociation or a vertical displacement fracture of the sacral body and without an anterior pelvic ring fracture. The patient was laid on a radiolucent table (Modular Table System; Mizuho OSI, California, USA) under general anesthesia. One or two folded blankets were put underneath the buttock to lift the patient off the table. The elevated height was adjusted depending on the pelvic deformity and patient’s physique. Reduction was carried out using either the open or closed method. Typically, closed reduction was attempted as the initial approach. However, if there was poor fracture reduction quality with closed reduction or there were additional fractures to be addressed, open reduction was performed. An intraoperative single-arm fluoroscopic intensifier (Ziehm Solo; Ziehm Imaging GmbH, Nuremberg, Germany) was used to visualize and diagnose pelvic discontinuity with serial pelvis projections, including AP, inlet/outlet, Judet, and sacral lateral views to ensure accurate screw position and reduction quality.

IS screws were inserted in the first sacral segment when possible, and TSIS screws were placed in the second sacral segment, rather than the first segment, when encountering sacral dysmorphism with a narrow corridor of the first sacral segment. To minimize technical errors, pre-drilling with a 2.0-mm K-wire was performed before inserting the 7.0-mm cannulated screw (Cannulated Screw 7.0 mm; Syntec Technology Co., Hsinchu, Taiwan). Oblique IS screws should be placed such that they are obliquely aligned posteriorly to anteriorly and inferiorly to superiorly while simultaneously crossing the sacroiliac joint as perpendicularly as possible. TITS screws were placed such that they crossed through the bilateral sacral ala and parallel to the ground when the patient was in the true supine position without radiolucent table tilting.

### Post-operative clinical outcome evaluation

Clinical outcomes were evaluated by an independent medical professional, and the assessments were conducted at 6 and 12 months post-operatively. The Postel Merle d'Aubigné and Majeed scores were utilized to assess the clinical outcomes. The Postel Merle d'Aubigné score assesses hip function and outcomes, focusing on pain, mobility, and walking ability; each of its components is rated on a scale of 0 to 6, with a total score of 18 classified as excellent, a score of 17 as very good, a score of 15–16 as good, a score of 13–14 as average, a score of 9–12 as poor, and a score  of < 9 as bad [22]. The Majeed score, on the other hand, evaluates outcomes and quality of life in patients with pelvic fractures. It includes factors such as pain, mobility, weight-bearing ability, daily activities, and emotional status, with each score ranging from 0 to 4 or 5; a total score  of ≥ 85 is classified as excellent, a score of 70–84 as good, a score of 55–69 as fair, and a score < 55 as poor [23]. Both scoring systems are valuable tools for clinicians to evaluate hip function following treatment and monitor patient progression over time.

### Statistical analysis

SPSS 25.0 (IBM Corp., Armonk, New York) was used for data processing and analysis. Categorical variables were compared using the chi-square test and Fisher’s exact test. The independent-samples *t* test was conducted for continuous data. Possible risk factors were analyzed by logistic regression.* P* < 0.05 (5%) was set as the cutoff value and considered statistically significant. The receiver operating characteristic (ROC) curve and Youden's J statistic were used to calculate the optimal PI cutoff value.

## Results

### Demographic data

In our study, a total of 85 patients with 135 screws were enrolled and extracted from our pelvic ring fracture database for analysis between January 2017 and June 2020. Patients were divided into two groups based on the percutaneous screw fixation technique: an IS screw group (65 screws) and a TITS screw group (70 screws). Then, we further divided them into four sub-groups based on the PI index value. To assess the PI values and the corresponding numbers of malpositioned screws, the cutoff PI values were set according to ROC analysis and Youden's J statistic to 49.85° in the IS screw group and 48.05° in the TITS screw group. Tables 1 and 2 summarize the demographic and baseline characteristics of all cases in detail.

**Table 1:** Patient characteristics of the iliosacral screw group

Variable	Small pelvic incidence (*N* = 41)	Large pelvic incidence (*N* = 24)	*p*
Basic data			
Male/female (*N*, ratio)	23/18	15/9	0.613
Age (years, mean ± SD)	33.27 ± 16.65	40.38 ± 18.60	0.117
Body mass index (kg/m^2^, mean ± SD)	23.01 ± 4.48	24.93 ± 4.39	0.097
Injury-related variables			
Associated injuries			
Brain (*N*)	9	3	0.511
Chest (*N*)	13	9	0.634
Abdomen (*N*)	11	3	0.222
Urogenital (*N*)	9	7	0.515
Extremities (*N*)	23	14	0.861
Spine (*N*)	5	0	0.149
AO/OTA classification			0.330
B1 (*N*)	1	0	
B2 (*N*)	13	12	
B3 (*N*)	18	5	
C1 (*N*)	4	5	
C2 (*N*)	3	0	
C3 (*N*)	2	2	
Tile classification			0.353
A (*N*)	1	0	
B (*N*)	31	17	
C (*N*)	9	7	
Young–Burgess classification			0.699
APC (*N*)	23	11	
LC (*N*)	16	9	
VS (*N*)	2	4	
Acetabular fracture (*N*)	7	6	0.441
Morel Lavallee (*N*)	3	6	0.066
ISS (mean ± SD)	24.20 ± 12.90	17.79 ± 12.92	0.058
NISS (mean ± SD)	26.73 ± 13.73	23.75 ± 16.46	0.436
Open fracture (*N*)	4	2	1.000
Surgery-related variables			
Injury-to-surgery interval (days, mean ± SD)	8.05 ± 4.04	8.25 ± 6.46	0.882
Surgical position			
Supine/prone (*N*, ratio)	30/11	16/8	0.578
Open/closed reduction (*N*, ratio)	13/28	7/17	0.830

*N* number, *SD* standard deviation, *m* meters, *kg* kilograms, *AO/OTA* Arbeitsgemeinschaft für Osteosynthesefragen/Orthopaedic Trauma Association, *ISS* Injury Severity Score, *NISS* New Injury Severity Score

**Table 2:** Patient characteristics of the trans-iliac trans-sacral screw group

Variable	Small pelvic incidence (*N* = 37)	Large pelvic incidence (*N* = 33)	*p*
Basic data			
Male/female (*N*, ratio)	22/15	15/18	0.241
Age (years, mean ± SD)	39.14 ± 19.16	44.52 ± 19.02	0.243
Body mass index (kg/m^2^, mean ± SD)	23.33 ± 4.36	23.44 ± 4.37	0.913
Injury-related variables			
Associated injuries			
Brain (*N*)	9	4	0.230
Chest (*N*)	9	11	0.405
Abdomen (*N*)	8	4	0.353
Urogenital (*N*)	13	5	0.056
Extremities (*N*)	22	18	0.678
Spine (*N*)	2	0	0.494
AO/OTA classification			0.279
B2 (*N*)	15	17	
B3 (*N*)	13	8	
C1 (*N*)	5	7	
C2 (*N*)	3	0	
C3 (*N*)	1	1	
Tile classification			0.477
B (*N*)	28	25	
C (*N*)	9	8	
Young–Burgess classification			0.323
APC (*N*)	14	11	
LC (*N*)	22	18	
VS (*N*)	1	4	
Acetabular fracture (*N*)	8	6	0.719
Morel Lavallee (*N*)	4	5	0.726
ISS (mean ± SD)	22.65 ± 9.46	15.47 ± 11.23	0.005*
NISS (mean ± SD)	25.57 ± 9.85	20.06 ± 14.93	0.072
Open fracture (*N*)	5	2	0.434
Surgery-related variables			
Injury-to-surgery interval (days, mean ± SD)	9.27 ± 5.28	9.24 ± 11.72	0.990
Surgery position			
Supine/prone (*N*, ratio)	31/6	24/9	0.260
Open/closed reduction (*N*, ratio)	7/30	6/27	0.937

*N* number, *SD* standard deviation, *m* meters, *kg* kilograms, *AO/OTA* Arbeitsgemeinschaft für Osteosynthesefragen/Orthopaedic Trauma Association, *ISS* Injury Severity Score, *NISS* New Injury Severity Score, * p value < 0.05

In both the IS and TITS screw groups, the patient’s demographic characteristics were comparable, with no significant differences despite the small and large PI values. In all groups, the primary injury mechanism was motor vehicle accident (*n* = 74), followed by fall (*n* = 23) and crush injuries (*n* = 22). Given the various types of pelvic fracture, the most common fracture pattern was AO/OTA classification B (B2.3 in the IS screw group, *n* = 16; B2.1 in the TITS screw group, *n* = 18), Tile classification B (B3 in the IS screw group, *n* = 22; B2 in the TITS screw group, *n* = 29), and Young–Burgess classification APC II (*n* = 25) and LC 1 (*n* = 25) in the IS and TITS screw groups, respectively. To minimize soft tissue damage, closed reduction with percutaneous screw fixation was the primary surgical approach. Considering that the majority of the high-energy fracture patients might also have suffered a concurrent anterior pelvic ring fracture, acetabular fracture, and/or other traumatic injuries, our surgical approach favored performing the procedure with patients in the supine position. However, in situations where patients had mild concurrent injuries and more complex posterior pelvic sacroiliac fractures, the prone position was adopted for better access during surgery. Ultimately, the decision regarding the surgical position was based on the surgeon's experience and preference. In our institute, instead of multiple-staged operations for patients, simultaneous fixations were performed in a single-stage surgery for both anterior and posterior pelvic ring fractures when the patient’s condition permitted. All patients underwent the surgery successfully without major iatrogenic complications, such as neurovascular, intra-abdominal, or genitourinary organ injuries.

### Radiographic outcomes

The radiographic parameters were measured pre- and postoperatively and documented as listed in Tables 3 and 4. The Matta/Tornetta and Lefaivre criteria were used to verify the quality of the reduction, and most patients presented acceptable reduction quality after the surgery. Interestingly, patients with smaller PI values were found to be significantly correlated with sacral dysmorphism (*p* = 0.027 and 0.003 in the IS and TITS screw groups, respectively). The IS screws were usually inserted at the level of S1 (96.92%), and the TITS screws at the level of S2 (74.29%). The screw malposition rates were 3.07% and 22.86% in the IS and TITS screw groups, respectively. In addition, patients with larger PI values were at significantly increased risk of screw malposition in the TITS screw group (*p* = 0.049), but such an association was not found for the IS screw group. The ADIO measurements of the IS and TITS screw groups showed no significant difference between patients with different PI values in both the axial and coronal views.

**Table 3:** Radiographic data and clinical outcomes of the iliosacral screw group

Variable	Small pelvic incidence (*N* = 41)	Large pelvic incidence (*N* = 24)	*p*
Pelvic incidence (degrees, mean ± SD)	42.81 ± 4.30	54.83 ± 5.38	< 0.001*
Sacral slope (degrees, mean ± SD)	33.34 ± 5.36	40.76 ± 5.28	< 0.001*
Pelvic tilt (degrees, mean ± SD)	9.47 ± 5.51	14.06 ± 4.83	0.001
Sacral dysmorphism (*N*)	16	3	0.027*
Reduction quality			
Matta/Tornetta criteria			0.406
Excellent (*N*)	19	9	
Good (*N*)	17	9	
Fair (*N*)	5	6	
Matta/Tornetta vertical (mm, mean ± SD)	6.71 ± 4.60	7.21 ± 4.45	0.669
Lefaivre rotational (mm, mean ± SD)	8.88 ± 7.79	12.53 ± 7.06	0.064
IS screw level			
S1 (*N*)	39	24	0.527
S2 (*N*)	2	0	0.527
IS screw direction			
Right/left (*N*, ratio)	21/20	10/14	0.457
IS screw length (mm, mean ± SD)			
IS screw ADIO on mpCT			
Coronal (degrees, mean ± SD)	6.45 ± 3.93	5.48 ± 3.03	0.301
Axial (degrees, mean ± SD)	9.31 ± 6.48	9.28 ± 7.62	0.984
IS screw perforation (*N*)	0	2	0.133
Perforation into			
Foramen/canal (*N*, ratio)	0/0	1/1	0.172
Perforation grading			0.172
Grade 1 (*N*)	0	1	
Grade 2 (*N*)	0	1	
Revision (*N*)	0	0	
Postel Merle d’Aubigne score			
At 6 months (mean ± SD)	10.68 ± 2.62	11.00 ± 2.25	0.622
At 1 year (mean ± SD)	14.66 ± 2.65	15.00 ± 2.21	0.597
Majeed score			
At 6 months (mean ± SD)	53.71 ± 14.36	57.96 ± 13.96	0.249
At 1 year (mean ± SD)	73.49 ± 12.89	75.08 ± 11.39	0.617

*N* number, *SD* standard deviation, *IS* Iliosacral, *ADIO* angle deviated from ideal orientation, *mpCT* multi-planar computed tomography, * *p* value < 0.05

**Table 4:** Radiographic data and clinical outcomes of the trans-iliac trans-sacral screw group

Variables	Small pelvic incidence (*N* = 37)	Large pelvic incidence (*N* = 33)	*p*
Pelvic incidence (degrees, mean ± SD)	41.61 ± 5.27	56.18 ± 6.70	< 0.001*
Sacral slope (degrees, mean ± SD)	31.71 ± 5.73	42.25 ± 5.97	0.001*
Pelvic tilt (degrees, mean ± SD)	9.90 ± 5.10	13.93 ± 4.81	< 0.001*
Sacral dysmorphism (N)	18	5	0.003*
Reduction quality			
Matta/Tornetta criteria			0.969
Excellent (*N*)	20	17	
Good (*N*)	11	10	
Fair (*N*)	6	6	
Matta/Tornetta vertical (mm, mean ± SD)	6.68 ± 6.00	6.01 ± 4.79	0.609
Lefaivre rotational (mm, mean ± SD)	8.03 ± 5.78	9.91 ± 6.39	0.200
TITS screw level			
S1 (*N*)	4	12	0.011*
S2 (*N*)	31	21	0.054
S3 (*N*)	2	0	0.494
TITS screw direction			
Right/left (*N*, ratio)	18/19	19/14	0.455
TITS screw length (mm, mean ± SD)	122.84 ± 8.46	123.03 ± 6.37	0.916
TITS screw ADIO on mpCT			
Coronal (degrees, mean ± SD)	1.90 ± 1.81	2.28 ± 2.63	0.478
Axial (degrees, mean ± SD)	3.09 ± 2.59	3.04 ± 3.30	0.941
TITS screw perforation (*N*)	5	11	0.049*
Perforation into			
Foramen/canal (*N*, ratio)	0/5	5/6	0.034*
Perforation grading			0.188
Grade 1 (*N*)	0	1	
Grade 2 (*N*)	3	4	
Grade 3 (*N*)	2	6	
Revision (*N*)	1	0	1.000
Postel Merle d’Aubigne score			
At 6 months (mean ± SD)	10.68 ± 2.62	11.00 ± 2.25	0.622
At 1 year (mean ± SD)	14.66 ± 2.65	15.00 ± 2.21	0.597
Majeed score			
At 6 months (mean ± SD)	53.71 ± 14.36	57.96 ± 13.96	0.249
At 1 year (mean ± SD)	73.49 ± 12.89	75.08 ± 11.39	0.617

*N* number, *SD* standard deviation, *TITS* trans-iliac trans-sacral, *ADIO* angle deviated from ideal orientation, *mpCT* multi-planar computed tomography, * *p* value < 0.05

The results of the logistic regression analysis are listed in Table 5. A larger PI value was confirmed as a significant risk factor for screw malposition in the TITS screws group, with *p* = 0.010. Interestingly, even with radiographic findings of screw malposition, no correlation with poor clinical outcomes or complications was found; even with a grade 3 perforation, no patient required revision surgery for screw adjustment. Among all the patients with grade 3 screw perforation, postoperative neurologic function could not be determined in one patient with a brain injury, and we decided to apply the utmost caution by removing the malpositioned screw in a concurrent debridement surgery for wound dehiscence to prevent potential undiagnosed neurological conditions.

**Table 5:** Logistic regression of the risk factors for screw perforation in the trans-iliac trans-sacral screw group

Predictor	Adj. OR (95% CI)	*p* value
Large/small pelvic incidence^#^	8.17 (1.67–40.01)	0.010*
Body mass index	1.08 (0.93–1.26)	0.306
Open/closed reduction	11.11 (0.38–325.73)	0.162
Supine/prone surgery position	0.12 (0.004–3.66)	0.223
Sacral dysmorphism	4.27 (0.94–19.33)	0.060

^#^ Large: ≥ 48.05°; small: < 48.05°

*OR* odds ratio, *%* percentage, *CI* confidence interval, *Adj* adjusted, * *p* value < 0.05

### Post-operative clinical outcomes

At 6 and 12 months post-operatively, the assessment using the Postel Merle d'Aubigné and Majeed scores revealed acceptable results for all four groups (Tables 3 and 4). At 6 months, patient outcomes were rated as poor according to the Postel Merle d'Aubigné score and poor to fair according to the Majeed score. However, a marked improvement in scores was noted at 12 months, with the majority of the outcomes ranging from average to good in both scoring systems. No statistically significant difference was observed between larger and smaller PI values within the IS and TITS groups. Overall, the patients expressed satisfaction with their improved outcomes.

## Discussion

Pelvic sacroiliac complex fractures are mostly caused by high-energy trauma, mostly in motor vehicle accidents. The percutaneous surgical approach is a popular method for restoring the pelvic anatomy and securing rigid fixation. Both IS and TITS screw fixation are excellent percutaneous fixation methods for surgical stabilization. However, due to the complexity of the sacroiliac structure, accurate screw placement with minimal soft-tissue dissection is highly technically demanding. Our study showed that a percutaneous approach provided rigid internal fixation with good reduction quality and clinical outcomes. However, there was a potential high risk for screw malposition while inserting the TITS screws. In addition, patients with larger PI values were found to be at significantly higher risk for TITS screw malposition.

Accurate screw placement remains of paramount importance, as it is related to pelvic construct stability and optimal outcomes. The screw malposition rate varies over a wide range (0–58.8%) in the published literature [24–27]. Our study demonstrated a malposition rate of 3.07% and 22.86% in the IS and TITS screw groups, respectively. Even though our results were comparable with previous studies, the high malposition rates raised concerns about neurovascular injury and needed to be addressed.

**Fig. 1:**
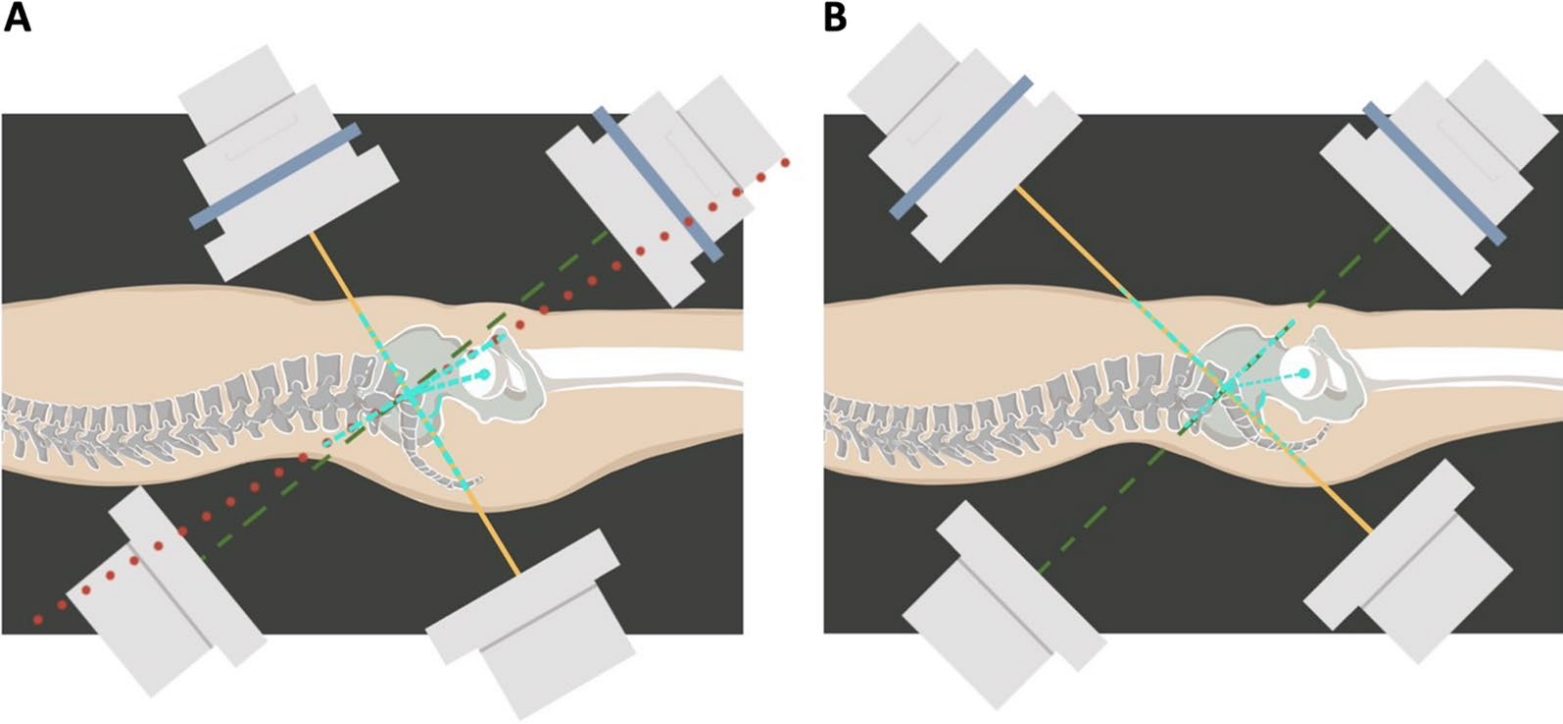
**A** With larger PI values, a greater fluoroscope cephalad tilt and a more horizontal projector placement were required. However, it was difficult to steer the fluoroscope to an appropriate angle of trajectory to get an optimal and clear pelvic outlet image due to hindrance from the radiolucent table or the patient’s thigh. When setting the pelvic inlet projection, the beamer of the single-arm fluoroscopic intensifier had to be aligned more perpendicularly with respect to the patient. The radiation could pass through excessive abdominal and buttock fat and bowel flatus, which could further interfere with and degrade the resolution of the images. **B** With smaller PI values, it is easier to position the fluoroscope to project the appropriate cephalad and caudal tilt angles.* Solid ine* caudad tilt of the fluoroscope,* dashed line* cephalad tilt of the fluoroscope,* dotted line* ideal cephalad tilt of the fluoroscope,* dashed-dotted line* PI angle,* PI* pelvic incidence

After analyzing the associated radiographic parameters, we found that the values of PI had a significant influence on the screw malposition rate in TITS screw fixation patients. In the IS screw fixation group, a 0% screw perforation rate was observed in patients with smaller PI values, compared to 8.33% in patients with larger PI values (*p* = 0.133). Interestingly, in the TITS screw group, the screw malposition rate was significantly lower in patients with smaller PI values (13.51%) when compared to those with larger PI values (33.33%; *p* = 0.049). This result can be explained by the difficult positioning of an intraoperative fluoroscope to obtain clear visualization of pelvic inlet and outlet projections in said patients. In general, in order to acquire good-quality images of inlet and outlet projections, the central ray projects at an angle 45° cephalic and 45° caudal to best demonstrate the pelvic ring configuration, and the projection angle might require minor adjustment to account for anatomical variations among individuals in order to optimize surgical outcomes. Ricci et al. found that, to achieve the intraoperative visualization of special bone corridors, an average caudal tilt of 21° of inlet angle was needed to profile the S1 anterior body; meanwhile, the average cephalad tilt outlet angle was 63° perpendicular to the S1 vertebral body and 57° for the S2 level [28]. Typically, patients with larger PI values require a greater cephalad tilt of the fluoroscope. With a horizontal fluoroscope placement, a clear pelvic outlet image can be obtained with the superimposed anterior and posterior rings of the first and second sacrum segments. The optimal images were often difficult to acquire when the fluoroscope trajectory was hindered or blocked by excess fatty tissue in the abdomen or thighs. Therefore, the inability to steer the fluoroscope to achieve an appropriate angle of trajectory might lead to a higher frequency of malpositioned TITS screws in patients with larger PI values (Fig. 1).

The PI was defined as the angle between the line perpendicular to the sacral endplate at its midpoint and the line connecting this point to the center of the femoral heads [10, 29]. In a given individual, this value remains constant after puberty and unchanged in any posture [30]. Moreover, PI values show a wide distribution range in the normal population. Abola et al. reported that a larger PI value was significantly correlated with a highly angulated and curved sacrum and corresponded to a more linear sacroiliac joint and narrower sacral alae in the sagittal plane [31]. Coudert et al. also described a similar finding in the horizontal plane and found that the articular surface orientation of the sacroiliac joint had a significant correlation with the PI value [32]. In our study, patients with larger PI values were found to have a significantly increased risk of screw malposition in the TITS screw group (*p* = 0.049), with logistic regression analysis also confirming that larger PI values were a significant risk factor in the TITS group (*p* = 0.010). We found that, even with an oblique osseous pathway of the sacral isthmus when inserting the IS screw, the surgeon was able to modify the screw trajectory while determining the safety zone and to make it slightly deviate from being perpendicular to the sacroiliac joint. This resulted in fewer patients with screw malposition.

Sacral dysmorphism is a common anatomical variant, which was first characterized as collinearity, mammillary processes, noncircular and misshapen anterior first sacral neuroforamina, and residual S1/S2 disc space by Routt et al. on plain radiographs [14]. In addition, Kaiser et al. used quantitative characteristics to define sacral dysmorphism, including coronal and axial angulation of the first sacral osseous corridor and the anatomic variation of the sacrum that prevents safe trans-sacral screw placement [16]. In our study, sacral dysmorphism was identified in 31.11% of patients, and we observed that it was significantly more common in patients with smaller PI values in both the IS and TITS screw fixation groups (*p* = 0.027 and 0.003, respectively). When sacral dysmorphism is present, an oblique and angulated trajectory makes screw placement difficult and demanding, even for experienced surgeons. However, no correlation was found between sacral dysmorphism and screw malposition rate in our study. This lack of correlation can be explained by the frequent insertion of IS screws at the level of the first sacral segment S1 (96.92%) in our study, while TITS screws were often placed at the level of the second sacral segment, S2 (74.29%). In the preoperative planning of IS screw placement, it was easier to modify the screw trajectory angle at the upper sacral segment in patients with sacral dysmorphism. Moreover, the second sacral segment was less influenced by the sacral dysmorphism, which should be considered when aiming for safe TITS screw placement.

Considering the close proximity of several neurovascular structures, the potential risk of neurovascular bundle damage due to screw malposition is a major concern in IS and TITS screw osteosynthesis procedures. Published studies have reported neurovascular complication rates ranging from 0% to 3.2% [25, 26, 33]. The exiting sacral nerve roots, descending lumbar nerve roots, as well as the iliac vessels and their distal branches are all critical structures that should be recognized to avoid any iatrogenic injury. Regarding the concerns about screw malposition, there is limited literature available that addresses specific scenarios in which screw removal or revision surgery is valid. Remiger et al. did not report screw removal in their cases [34], while Routt et al. suggested that removal should only be performed in cases of screw breakage or dislodgement [9]. A review from Yücel et al. highlighted that removal may be reasonable in patients with an infection or persistent local pain or neurological symptoms caused by screw malposition [35]. In our study, despite the screw malposition rates of 3.07% and 22.86% in the IS and TITS screw groups, respectively, no perioperative vascular injuries were detected, and none of the patients reported experiencing any post-operative neurological symptoms or deficits. The only patient who underwent revision surgery had a concurrent brain injury, which hindered the comprehensive assessment of post-operative neurologic function. Therefore, we decided to remove the malpositioned screw during debridement surgery for wound dehiscence in the hope of preventing potential undiagnosed neurological conditions.

Whether to perform reduction with precise and quick percutaneous screw placement with the patient in the supine or prone position is still under debate. Initially, IS and TITS screw fixation procedures were performed with the patient in the prone position, but in 1992 Routt et al. introduced the use of the supine position when performing the surgery [36]. The prone position allowed the surgeons to focus on the posterior pelvic sacroiliac complex fracture. However, that position had some limitations, as the pelvic deformity could be accentuated by gravity, which could make reduction more difficult. Second, it could induce an increase in intra-abdominal pressure as a consequence of the prone positioning [37, 38]. In contrast, performing the surgery in the supine position had several advantages in terms of the pelvic ring and hemodynamic stability. Moreover, to ensure a smooth workflow, the supine position allowed surgeons to simultaneously perform other procedures for other concomitant injuries, such as intra-abdominal/genitourinary organ injuries or limb fractures, without changing position and re-draping the surgical fields. In our study, 74.81% of patients underwent the surgery in the supine position (70.77% and 78.57% in the IS and TITS screw groups, respectively). In the supine position, when setting the pelvic inlet projection, the beamer of the single-arm fluoroscopic intensifier had to be aligned more perpendicularly with respect to the patient for larger PI values, and this could result in the radiation passing through excessive abdominal fat and bowel flatus, which could interfere with and degrade the resolution of the images. This interference may make large PI values a risk factor for screw malposition (Fig. 1).

**Fig. 2:**
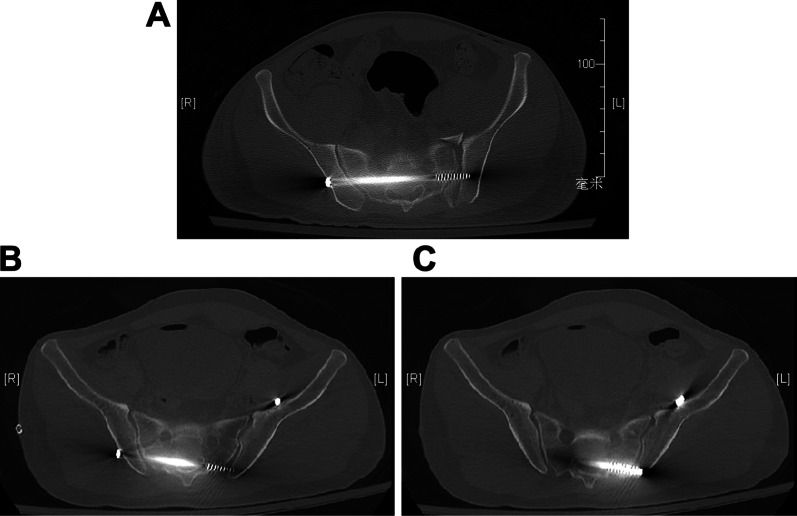
**A** TITS screw placement at the S2 level was successfully executed along the intended projection. **B**, **C** A TITS screw placed at the S2 level was found to be malpositioned within the spinal canal. *TITS* trans-iliac trans-sacral

Our work had some limitations. First, the study design was a single-center, retrospective study with two groups, which were then divided into four sub-groups with small and unequal sample sizes, and this may have compromised the generalizability of our results. Second, all the procedures were performed by a single surgeon. With an improvement in surgical performance over time, a decrease in the screw malposition rate could be expected, which could affect surgical outcomes. Despite these inherent limitations, our work has important implications for surgeons performing TITS screw fixation in patients with large PI values, as preoperative surgical planning is paramount when attempting to reduce screw malposition and potential neurovascular complications (Fig 2).

## Conclusion

Percutaneous IS and TITS screw fixations are safe, effective, and minimally invasive procedures to treat posterior pelvic sacroiliac fracture. These techniques have become increasingly popular. Our study demonstrates that, for TITS screw fixation, larger PI values are a risk factor for screw malposition, highlighting their potential role in ensuring good surgical outcomes.

## Data Availability

All data and materials are available. The corresponding author can provide the datasets used and/or analyzed during the current study upon reasonable request.

## Abbreviations

ADIO: Angles deviated from ideal orientation
AO/OTA: Arbeitsgemeinschaft für Osteosynthesefragen/Orthopaedic Trauma Association
AP: Anterior–posterior
IS: Iliosacral
mpCT: Multi-planar computed tomography
PI: Pelvic incidence
ROC: Receiver operating characteristic
TITS: Trans-iliac trans-sacral
